# CREBZF expression and hormonal regulation in the mouse uterus

**DOI:** 10.1186/1477-7827-11-110

**Published:** 2013-12-10

**Authors:** Pengfei Lin, Fenglei Chen, Nan Wang, Xiangguo Wang, Xiao Li, Jinhua Zhou, Yaping Jin, Aihua Wang

**Affiliations:** 1 Key Laboratory of Animal Biotechnology of the Ministry of Agriculture, Northwest A&F University, Yangling, Shaanxi 712100, China; 2 College of Veterinary Medicine, Northwest A&F University, Yangling, Shaanxi 712100, China

**Keywords:** Embryo implantation, Mouse, Uterus, CERBZF, SMILE

## Abstract

**Background:**

CREBZF is a member of the mammalian ATF/CREB family of the basic region-leucine zipper (bZIP) transcription factors. Two isoforms of CREBZF have been identified from the alternative usage of initiation codons, SMILE (long isoform of CREBZF) and Zhangfei (short isoform of CREBZF). Until recently, the physiological function of CREBZF in mammalian reproductions has not been reported.

**Methods:**

Multiple techniques were performed to investigate the spatiotemporal expression and hormonal regulation of the CREBZF gene in the mouse uterus and its role in embryo implantation.

**Results:**

Zhangfei was not detected in the mouse uterus. SMILE immunostaining was mainly expressed in the uterine luminal and glandular epithelium, and the expression levels of both SMILE mRNA and protein gradually decreased from days 1–3 of pregnancy, peaked on day 4, and then declined again on day 6. On day 5 of pregnancy, SMILE protein expression was detected only in the luminal epithelium at implantation sites compared with the expression at inter-implantation sites. SMILE protein was not detected in decidual cells from days 6–8 of pregnancy or artificial decidualisation. Furthermore, SMILE protein was not detected in the mouse uterus on days 3–6 of pseudopregnancy, and SMILE expression was also induced in the delayed-implantation uterus, indicating that the presence of an active blastocyst was required for SMILE expression at the implantation site. Oestrogen significantly stimulated SMILE expression in the ovariectomised mouse uterus. In addition, in cycling mice, high levels of SMILE protein and mRNA expression were also observed in proestrus and oestrus uteri.

**Conclusions:**

Taken together, these results suggested that SMILE expression was closely related to mouse implantation and up-regulated by oestrogen.

## Background

Successful implantation is dependent on the intricate genetic and molecular signalling dialogue between the receptive uterus and active embryo [1]. A better understanding of the molecular events underlying the regulation of embryo implantation may improve the ability to treat infertility. To date, although many molecular modulators have been identified during the implantation period, the precise molecular mechanism underlying embryo implantation is still unknown.

CREBZF is as a member of the CREB (cAMP-response-element-binding protein)/ATF (activating transcription factor) family of basic region-leucine zipper (b-ZIP) transcription factors [2]. Previously studies have found that CREBZF gene expression produces two isoforms, CREBZF-L (long isoform of CREBZF, also known as SMILE) and CREBZF-S (short isoform of CREBZF, which was previously designated as Zhangfei or ZF) [3]. However, unlike other b-ZIP proteins CREBZF lacks the ability to bind any of the consensus recognition elements for b-ZIP proteins and cannot activate promoters containing these motifs [2,4]. Instead, CREBZF requires heterodimerisation with other factors to bind target promoters and to regulate downstream genes, which may include VP16 [5], Luman (or CREB3, L-ZIP) [6], Activating transcription factor 4 (ATF4, or CREB2) [7], X-box-binding protein-1 (XBP1) [8] and tropomyosin-related kinase A (trkA) [9,10]. A potential reason for the inability of CREBZF to recognise these promoters may be the absence of a critical asparagine residue in its basic domain, which forms the DNA-recognition motif, NxxAAxxCR, in all b-ZIP proteins [2,4].

Zhangfei was first identified via its interaction with the Herpes Simplex Virus-1 (HSV-1)-related cellular protein Host Cell Factor 1 (HCF-1) and has been proposed to play a role in inhibiting the replication of the herpes simplex virus [2,5]. Recent studies have shown that Zhangfei could potentially play an important role in the mammalian unfolded protein response (UPR) [6-8]. Zhangfei might activate apoptosis in ONS-76 medulloblastoma cells via the NGF/TrkA pathway [10,11]. Currently, the precise physiological function of SMILE remains unclear. Recently, SMILE has been reported as a novel transcriptional co-regulator of a variety of nuclear receptors, including oestrogen receptors, glucocorticoid receptor, constitutive androstane receptor, hepatocyte nuclear factor 4α and oestrogen receptor-related receptor γ [3,12,13]. In another study, SMILE played an important role in the development of beta cell dysfunction induced by glucolipotoxicity [14]. Zhangfei exhibits a liver- and cell-type-specific expression pattern; however, SMILE is expressed ubiquitously in mouse tissues and tumour-derived cells [3]. Thus, it is certainly possible that the two isoforms exhibit different regulatory functions in specific cellular contexts. However, until recently, the physiological function of CREBZF in mammalian reproduction has not been reported. To explore the potential function of CREBZF during pregnancy, we sought to investigate in detail the expression and regulation of CREBZF mRNA and protein in the mouse uterus during the oestrous cycle and peri-implantation period.

## Methods

### Animals and treatments

The experimental use of mouse for this study was performed according to the Committee for the Ethics on Animal Care and Experiments in Northwest A&F University. Mature mice (Kunming White outbred strain, 8-to 10-wk-old) were purchased from the laboratory animal centre of Xi’An JiaoTong University and housed at a temperature- (24+/−2°C) in a light-controlled room (12 h light: 12 h darkness) with free access to food and water.

Oestrous cycles were tracked by performing daily vaginal smears, and only those mice that demonstrated a regular 4-day oestrous cycle were used for these experiments. Mouse models of early pregnancy, pseudopregnancy, delayed implantation and activation, artificial decidualisation and hormonal treatment were produced as described in our previous reports [15,16]. Eight mice were used in each stage or treatment in this study. To confirm the reproducibility of the results, each sample was analysed in triplicate. The entire uterus was collected immediately following sacrifice by cervical dislocation, and half of each uterus was immediately processed for immunohistochemistry, whereas the remaining half was frozen in liquid nitrogen for further extraction of total RNA and protein.

### Embryo collection

Female mice were superovulated via an intraperitoneal injection of 5 IU of pregnant mare’s serum gonadotrophin (PMSG, Ningbo Sansheng Pharmaceutical Co., Ltd. China) at 4:00 pm followed by human chorionic gonadotrophin (hCG, Ningbo Sansheng Pharmaceutical Co., Ltd. China) 48 hr later. Treated female mice were mated with fertile males of the same strain to induce natural pregnancy. Embryos at the zygote, 2-cell and 4-cell stage were collected from oviducts at 24 hr, 46 hr and 54 hr, respectively. However, the 8-cell stage embryo, morula and blastula were collected from the uterine horns at 68 hr, 72 hr and 96 hr, respectively. A total of 160 mice were used for embryo collection and 35–40 pooled embryos per developmental stage were analyzed in each group. Three independent experiments were performed.

### RNA extraction, cDNA synthesis and real-time quantitative PCR

Total RNA was extracted from the uterine horns and embryo using Trizol (Invitrogen, Inc., Carlsbad, CA, USA) according to the manufacturer’s instructions. DNase (TaKaRa Bio, Inc., Dalian, China) was used to remove genomic DNA contamination prior to RT. Extracted RNA was dissolved in diethylpyrocarbonate (DEPC)-treated water, and the RNA concentration and purity were estimated by reading the absorbance at 260 and 280 nm on a spectrophotometer (Eppendorf, Inc., Hamburg, Germany). The cDNAs were synthesised using PrimeScript^TM^ RT reagent Kit (TaKaRa Bio, Inc., Dalian, China) according to the manufacturer’s instructions. The final volume of the reaction was 20 μl, including 800 ng of total RNA. The reverse transcription product was stored at −20°C.

The GenBank accession number of the mRNA, primer sequences and annealing temperatures are listed in Table 1. Real-time PCR was performed using three biological replicates and technical triplicates/duplicates of each cDNA sample in the LightCycler system (iQ5, Bio-Rad Laboratories, Inc., Hercules, USA) using SYBR® Premix Ex Taq^TM^ II Kit (TaKaRa Bio, Inc., Dalian, China), according to the manufacturer’s protocol. Each PCR reaction (total volume of 20 μl) consisted of 2 μl reverse transcription product, 0.8 μl of each 10 μM forward and reverse primer, 10 μl SYBR® Premix Ex Taq^TM^ II, and 6.4 μl RNase-free water. The cycling conditions included a denaturation step at 95°C for 30 sec, followed by 45 PCR cycles of 95°C for 5 sec and 60°C for 20 sec. A melting curve analysis was performed at the end of each PCR program to exclude the formation of nonspecific products. Gene mRNA quantifications were performed using the 2^-△△Ct^ method and the amount of transcripts in each sample was normalised using RPLP0 and GAPDH as the internal control gene to correct for differences in the amount of cDNA used [17,18].

**Table 1:** The primer sequences used for real-time quantitative PCR

**Target gene**	**GenBank accession no.**	**Primer sequence**	**Product size (bp)**	**Annealing temperature (°C)**
*SMILE*	NM_145151.2	AF: 5*'*-TAATCGGCTCAAGAAGAAGG-3*'*	144	60
		AR: 5*'*-CGTAGGTAGCGACTCTCC-3*'*		
*RPLP0*	NM_007475	AF:5*'*- GGACCCGAGAAGACCTCCTT-3*'*	85	60
		AR:5*'*- GCACATCACTCAGAATTTCAATGG-3*'*		
*GAPDH*	NM_008084	AF:5*'*- TCACTGCCACCCAGAAGA-3*'*	186	60
		AR:5*'*- GACGGACACATTGGGGGTAG-3*'*		

### Immunohistochemistry

Uterine tissues were fixed in 4% (v/v) paraformaldehyde (Sinopharm Chemical Reagent Co., Ltd, Shanghai, China) in phosphate-buffered saline (PBS; pH 7.4) for 24 hr, dehydrated through a graded ethanol series, and embedded in paraffin. Five μm-thick sections were mounted onto glass slides that were precoated with poly-L-Lysine solution (Sigma, St. Louis, MO, USA) and incubated overnight at 37°C. After dehydration, the samples were placed in citrate buffer (pH 6.0), and antigen retrieval was performed by treating the samples (twice) in a microwave oven at 750 W for 5 min. The slides were then washed in PBS. Sections were pretreated with 0.3% (v/v) H_2_O_2_ in methanol to quench endogenous peroxidase activity. After several washes with PBS, the sections were incubated with 10% rabbit serum for 30 min at 37°C. Following blocking, the sections were incubated with goat anti-CREBZF polyclonal antibody (Santa Cruz, sc-49328; diluted 1:200 with PBS) for 12 hr at 4°C, washed with PBS, and incubated with biotinylated anti-goat IgG antibody (MaiXin-Bio Technology Co., Ltd., Fuzhou, China) for 1 hr at 37°C. Sections were washed three times with PBS, and then incubated with HRP-labelled streptavidin (SA-HRP) for 30 min at 37°C. Next, positive reactions were visualised using a diaminobenzidine (DAB)-peroxidase substrate (Sigma-Aldrich Co. LLC, Louis, MO, USA) and 30 sec counterstaining with haematoxylin. Negative control slides without the addition of primary antibody or substitution with an appropriate dilution of normal goat IgG was performed in parallel. Slides were imaged using a digital microscope (BA400, Motic, Wetzlar, Germany).

### Western blotting analyses

Proteins obtained from uterine tissues were extracted using the Total Protein Extraction Kit (Nanjing Keygen Biotech Co., Ltd., Nanjing, China) according to the supplier’s instructions. The total protein per sample was separated using 12% sodium dodecyl sulphate (SDS)-polyacrylamide gel electrophoresis (PAGE) followed by electrotransfer to polyvinylidene fluoride (PVDF) membranes (Millipore; Bedford, MA). The membrane was treated with blocking buffer (5% nonfat dried milk in Tris-buffered saline [TBS] containing 0.1% Tween 20) for 1 hr at room temperature and incubated overnight at 4°C with antibodies against CREBZF (Santa Cruz, sc-49328; 1:500 dilution) or β-actin (1:1,000; Beijing CWBIO Co., Ltd., Beijing, China) as a loading control. The membranes were washed three times with TBS, and then incubated with biotinylated anti-goat IgG antibody or biotinylated anti-mouse IgG antibody (1:5,000; MaiXin-Bio Technology Co., Ltd., Fuzhou, China) for 1 hr at room temperature. After washing, the membranes were incubated with SA-HRP for 30 min at room temperature. Finally, the immunoreactive bands were visualised by DAB staining (Sigma) at room temperature for 5 min, and the immunoreactive bands were imaged using a digital microscope (Tanon-4100, Tanon Science & Technology Co., Ltd., Shanghai, China) and densitometric analyses were processed with Quantity one v4.62 (Bio-Rad).

### Immunofluorescence

Embryos were fixed in 4% paraformaldehyde for 30 min at room temperature followed by permeabilising in 0.5% Triton X-100 in PBS. After washing in PBS, the embryos were treated with blocking solution of 1% bovine serum albumin in PBS for 1 hr, followed by incubation with goat anti-CREBZF polyclonal antibody (Santa Cruz, sc-49328; 1:500 dilution) at 4°C overnight. Embryos were then washed three times with PBS, and incubated for 1 hr at room temperature in a 1:500 dilution of Cy3-labelled donkey anti-goat IgG (Beyotime Institute of Biotechnology, Jiangsu, China). The nuclei were stained with 4,6-diamidino-2-phenylindole (DAPI). Embryos were viewed under a laser-scanning confocal microscope (A1R, NiKon). Additionally, negative control embryos per developmental stage were treated in the same manner as described above, but omitting the primary antibody or primary and secondary antibodies, respectively.

### Statistical analysis

All experiments were replicated at least three times for each group and the data were presented as the mean ± S.E.M. Data were analysed using ANOVA, followed by Fisher’s Least Significant Different Test (Fisher LSD) and Independent-Samples T test with SPSS software (Version 13.0; SPSS, Inc., Chicago, IL). Differences were considered significant when *P* < 0.05.

## Results

### CREBZF protein expression in the mouse uterus

**Figure 1:**
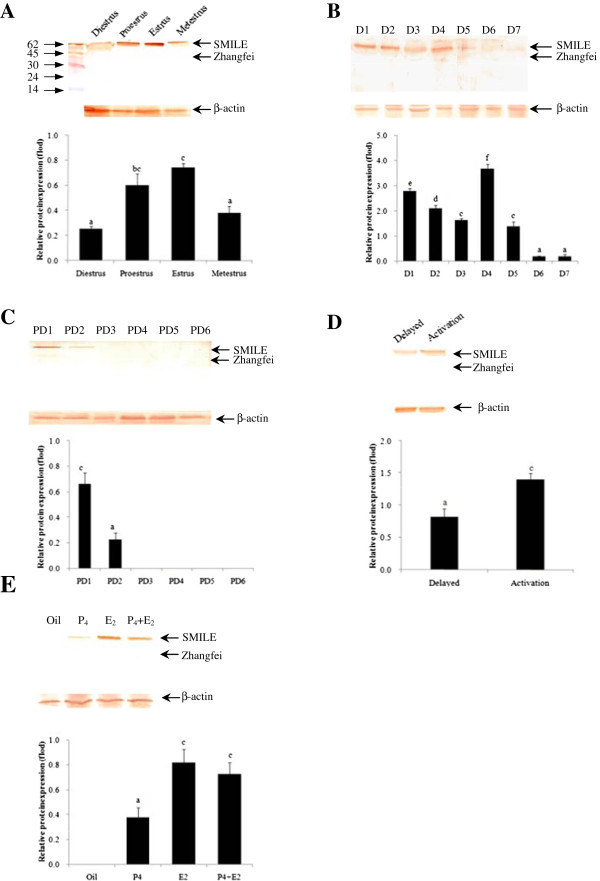
**Immunoblot analysis for CREBZF proteins in the mouse uterus during oestrous cycle (A), early pregnancy (B), pseudopregnancy (C), delayed implantation (D), and steroid hormonal treatments (E).** β-actin levels are shown as a loading control. The values represent the mean ± S.E.M. from three duplicates, and the bar bearing different superscript indicates a significant difference between the mean values (*P* < 0.05).

Changes in CREBZF protein expression in whole uterine protein extracts were detected using western blotting analyses. In all samples analysed, a single band at approximately 50–60 kDa was observed (Figure 1). Xie *et al*. [3] reported that SMILE and Zhangfei isoforms were apparent at molecular masses of 50–60 kDa and 40 kDa, respectively. These results indicated that SMILE was the predominant form in this study, whereas Zhangfei was not detected in the mouse uterus.

The positive control for SMILE and Zhangfei protein was analysed by western blotting using a CREBZF-specific antibody. Results are representative of at least three independent experiments with similar results. MCF7, human breast adenocarcinoma cell line. 293 T, human embryonic kidney cell line (Additional file 1: Figure S1).

### SMILE is expressed in the mouse uterus throughout the oestrus cycle

**Figure 2:**
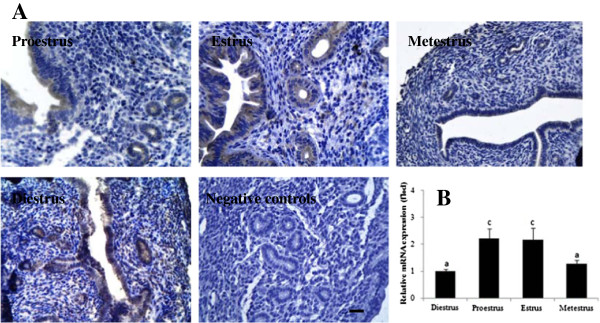
**Expression of SMILE in the mouse uterus during different phases of the oestrus cycle. (A)** Immunolocalisation of SMILE protein in the luminal and glandular epithelial cells of the uterus during the mouse oestrous cycle. Bars =25 μm. **(B)** Expression of SMILE mRNA in cycle uteri as detected using real-time PCR. The values represent the mean ± S.E.M. from three duplicates, and the bar bearing different superscript indicates a significant difference between the mean values (*P* < 0.05).

To determine whether there are differences in the expression and localisation of SMILE mRNA and protein during the oestrus cycle, SMILE mRNA and protein expression in the mouse uterine at proestrus, oestrus, metestrus and diestrus was detected using immunohistochemistry, real-time PCR and western blotting, respectively. We found that independent of the phase of the oestrus cycle, SMILE protein was specifically expressed in the endometrial epithelial cells of both the lumen and glands, while the expression was most prominent at the oestrus phase (Figure 2A). Real-time PCR and western blotting analysis also revealed that the levels SMILE mRNA and protein at oestrus were significantly higher compared to the levels from metestrus and diestrus, although these values were not significantly different compared with those obtained from the proestrus phase (*P* < 0.05, Figures 1A and 2B).

### SMILE expression during early pregnancy

**Figure 3:**
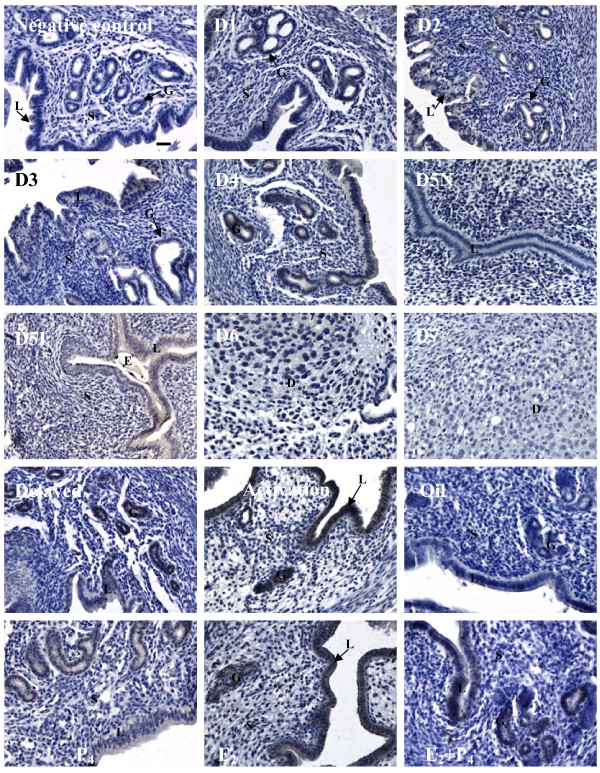
**Localisation of SMILE protein in the mouse uterus during early pregnancy, delayed and activated implantation and steroid hormonal treatments, examined by immunohistochemistry. D1**-**D7**, the sections were stained with CREBZF antibody for days 1–7 pregnant uterus; **D5I**, implantation sites on day 5 of pregnancy; **D5N**, inter-implantation sites on day 5 of pregnancy. L = luminal epithelium; G = glandular epithelium; S = stromal cells; D = decidual cells; E = embryo. Bars =25 μm.

SMILE protein was mainly localised in the luminal and glandular epithelium during days 1 to 5 of pregnant uteri, whereas the staining intensity on day 4 of pregnancy was significantly increased (Figure 3 D1-D5). Compared with the inter-implantation site, the positive signal was observed in the luminal epithelium at the implantation site on day 5 of pregnancy (Figure 3 D5N and D5I). No obvious immunostaining of the SMILE protein was detected in the decidual cells, and SMILE only presented in the remaining glandular epithelium from days 6–7 of pregnancy (Figure 3 D6 and D7). Moreover, no specific signal for SMILE protein was detected in the artificial decidualisation model, and SMILE mRNA expression also did not show a significant difference in the decidualised uterus compared with the control uterus (data not shown).

**Figure 4:**
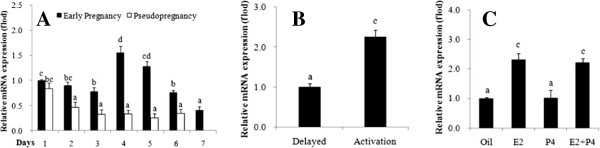
**Relative levels of SMILE mRNA in the mouse uterus during early pregnancy and pseudopregnancy. (A)**, delayed implantation **(B)** and steroid hormonal treatments **(C)** were assessed using real-time PCR. SMILE expression was normalised to RPLP0 and GAPDH. The values represent the mean ± S.E.M from three independent experiments. Bars with different letters are significantly different (*P* < 0.05).

Real-time PCR was also used to detect the levels of SMILE mRNA expression in the mouse uterus on days 1–7 of pregnancy (Figure 4A). SMILE mRNA levels were gradually decreased on days 1–3 of pregnancy and were significantly lower on day 3 compared to day 1. These levels then peaked on day 4 (*P* < 0.05, Figure 4A). However, the level of SMILE mRNA was significantly decreased in the mouse uterus as the decidua was developing from day 6 to 7 of pregnancy. Similar with the results obtained from real-time PCR, SMILE protein was gradually decreased from day 1 to day 3, but it was up-regulated in day 4 uteri and was nearly not detectable from day 6 to day 7 uteri (Figure 1B).

### SMILE expression during pseudopregnancy and activated implantation

In pseudopregnant mice, a basal level of SMILE staining was observed in luminal and glandular epithelium on days 1–2; however, no positive staining was observed in the uterus from day 3–6 (data not shown). Compared to early pregnancy, the levels of both SMILE mRNA and protein were lower on day 2 in pseudopregnant uteri (Figures 1C and 4A). As shown in Figure 3, SMILE immunostaining was abundantly expressed in activated implantation uteri, while its staining was weakly expressed in delayed implantation uteri. Moreover, significantly higher levels of SMILE mRNA and protein were also induced in the activated implantation uterus compared with the levels in the delayed uterus (*P* < 0.05, Figures 1D and 4B). Taken together, these observations strongly suggested that the presence of embryos affected SMILE localisation in these epithelial cells.

### Steroid hormonal regulation of SMILE expression

Because E_2_ and P_4_ are essential for mouse embryo implantation, ovariectomised mice were used to examine the effects of steroid hormones on CREBZF expression. We found no visible signal in the ovariectomised mice (Figure 3). In the P_4_-treated group, a low level of SMILE immunostaining was present in the luminal and glandular epithelium. However, E_2_ significantly induced the expression of SMILE protein both in the luminal and glandular epithelium. There was only a moderate level of SMILE expression in these cells via combined E_2_ and P_4_ treatment. To further confirm SMILE expression, real-time PCR and western blotting were also performed. No significant difference in SMILE mRNA expression was observed after an injection of P_4_ compared to that of oil treatment (Figure 4C). In contrast, the expression levels of SMILE mRNA and protein in the uterus were significantly stimulated by E_2_ treatment (*P* < 0.05, Figures 1E and 4C). After the combined injection of E_2_ and P_4_, the SMILE mRNA and protein expression in uterus were similar to those in the E_2_ treatment alone, respectively.

### Localisation of SMILE in mouse embryos during the peri-implantation period

**Figure 5:**
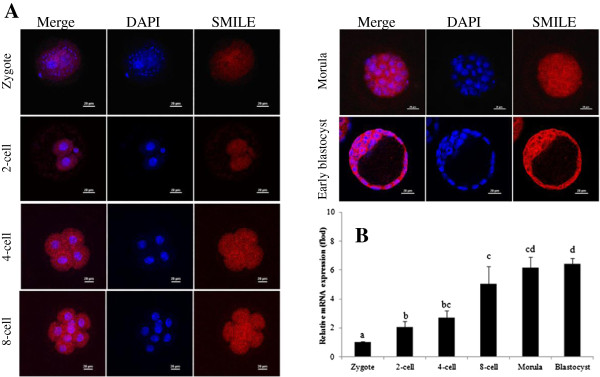
**Expression of SMILE mRNA and protein in the mouse embryos during pre-implantation. (A)** Immunofluorescence analyses of SMILE protein in embryos from the zygote to the blastocyst stage. The red colour represents SMILE staining, and the blue colour indicates nuclear staining. Bar = 20 μm. **(B)** Analysis of SMILE mRNA levels using real-time PCR in the mouse embryos. The values represent the mean ± S.E.M from three independent experiments. Bars with different letters are significantly different (*P* < 0.05).

To further study the role of SMILE in embryo implantation, we examined its localisation in the mouse embryos from the zygote to the blastocyst stage. These results demonstrated that SMILE was expressed at all stages of mouse pre-implantation embryos and was mainly localised in the cytoplasm and nucleus (Figure 5A). At the blastocyst stage, strong signals of SMILE protein were observed in the trophectoderm and the inner cell mass (Figure 5A). Furthermore, the levels of SMILE mRNA were gradually increased from the zygote to the blastocyst stage in the embryo (*P* < 0.05, Figure 5B).

## Discussion

This study mainly reported on the changes in the expression of two isoforms of CREBZF, as well as their cellular localisation throughout the oestrous cycle and peri-implantation period in the mouse uterus. These results indicated that SMILE was the predominant form and Zhangfei was not detected in the uterus, which was consistent with the expression pattern of SMILE in the male mouse prostate and testis [3]. Moreover, our results suggested that SMILE had a cycle-dependent expression in mouse uterus and was clearly localised in both luminal and glandular epithelial cells. A relatively higher expression of SMILE mRNA and protein at the oestrus phase accompanies the female as she reaches optimal sexual receptivity and ovulating time. Thus, we speculated that the elevated levels of SMILE might contribute to the induction of embryo earlier during development and implantation. Indeed, SMILE was localised in the cytoplasm and nucleus of mouse pre-implantation embryos, and the level of SMILE mRNA was gradually increased with embryo development. In addition, SMILE was expressed at high levels in the luminal and glandular epithelium at the proestrus and oestrus phase, and at basal levels in the metestrus and diestrus phase. These data also suggested that SMILE might be under cell-specific sex hormonal control in the mouse uterine epithelium and appeared to be important factors for implantation.

During pregnancy the uterus undergoes a series of programmed morphological changes, resulting in an extensive tissue reorganisation to accommodate the growing embryo [19]. The time from days 1 to 5 of pregnancy is critical for embryo implantation in mice, and days 4 to 5 of pregnancy is regarded as the implantation window. The embryo apposes and attaches to the uterine luminal epithelium, which is the initial step for a successful pregnancy [20]. The expression levels of SMILE mRNA and protein were gradually decreased in the mouse uterus from days 1 to 3, but were sharply up-regulated on day 4. Furthermore, the uterine expression of the SMILE protein in the luminal epithelium was predominantly localised at the implantation site on days 5 of pregnancy, demonstrating temporal and spatial specificity during the period of mouse embryo implantation. Taken together, these results suggested that SMILE might play an essential role in the initial attachment of a blastocyst to the uterine epithelia in the mouse. However, the up-regulation of SMILE was restricted to day 5 and was not expressed in decidualised cells on days 6–7 of early pregnancy or under artificial decidualisation, although SMILE was also highly expressed in glandular epithelial cells, which might not be important for decidualisation.

In the present study, SMILE was lowly expressed on days 1–2 in the pseudopregnant uteri and was not observed in the uterus from days 3–6 of pseudopregnancy, indicating that SMILE expression was dependent on the presence of the embryo. Moreover, SMILE mRNA and protein were highly expressed in the uterus when the delayed implantation was terminated by via treatment compared with the expression under conditions of delayed implantation. In addition, the localisation of SMILE protein in the trophectoderm and increased expression of SMILE mRNA and protein at the blastocyst stage might be a prerequisite for establishing contact between the blastocyst and uterine epithelium. These results suggested that SMILE expression in the mouse uterus required the presence of an active blastocyst during the peri-implantation period.

Implantation has long been known to be a steroid hormone-dependent process. The coordinated action of both E_2_ and P_4_ is necessary for the preparation and process of implantation in the mouse endometrium [21]. Because both E_2_ and P_4_ are essential for the induction of implantation in the mouse, we examined the hormonal regulation of SMILE expression. It has been reported that CREBZF isoforms regulated the inhibition of the transactivation of oestrogen receptors via a small heterodimer partner in a cell-type-specific manner, although CREBZF isoforms did not interact directly with oestrogen receptors [3]. CREBZF isoforms as a novel corepressor of nuclear receptors may interact with SIRT1 to regulate the transactivation of oestrogen receptor-related receptor γ [13]. SIRT1 plays an important role in the development of human uterine receptivity via inducing E-cadherin expression [22]. Our studies found that E_2_ could significantly up-regulate the expression and localisation of SMILE in the luminal epithelium and glandular epithelial cells in ovariectomised mice. In addition, P_4_ had no significant effects on SMILE expression in the ovariectomised mouse uterus. Similar hormonal regulation was also consistent with the spatiotemporal expression of SMILE mRNA and protein in the mouse uterus during the oestrous cycle in this study. High levels of E_2_ were observed in mice during proestrus and oestrus, decreasing to low levels during metestrus and diestrus [23]. During normal mouse pregnancy, higher levels of E_2_ were detected on day 1 and day 4, a surge in E_2_ on day 1 stimulated uterine epithelial cell proliferation and induced the expression of progesterone receptors. An increase in E_2_ levels on day 4 combined with the high P_4_, which further stimulated uterine stromal proliferation and induced the endometrial receptivity for the blastocyst to implant [21,24]. The results in ovariectomised mice, oestrous cyclic mouse in pregnant mice confirmed our supposition that the expression of SMILE in the peri-implantation uterus may be mainly modulated by E_2_.

## Conclusions

On the basis of these findings, we concluded that SMILE might play a potential role in mediating uterine receptivity during implantation and development of preimplantation embryos in mice. Moreover, SMILE expression in mouse uteri was regulated by active blastocysts and E_2_.

## Abbreviations

ATF: Activating transcription factor; ATF4: Activating transcription factor 4; b-ZIP: Basic region-leucine zipper; CREB: cAMP-response-element-binding protein; DAB: Diaminobenzidine; DEPC: Diethylpyrocarbonate; E2: Oestrogen; HCF-1: Host cell factor 1; hCG: human chorionic gonadotrophin; HSV-1: Herpes simplex virus-1; P4: Progesterone; PAGE: Polyacrylamide gel electrophoresis; PBS: Phosphate-buffered saline; PMSG: Pregnant mare’s serum gonadotrophin; PVDF: Polyvinylidene fluoride; SDS: Sodium dodecyl sulphate; TBS: Tris-buffered saline; trkA: tropomyosin-related kinase; UPR: Unfolded protein response; XBP1: X-box-binding protein-1.

## Competing interests

The authors declare that they have no competing interests with respect to the authorship and/or publication of this article.

## Authors’ contributions

PL and AW designed the experiments and drafted the manuscript. NW and JZ collected the uterine samples and embryos. FC, XW, and XL performed the experiments. YJ organised and supervised the project. All authors read and approved the final manuscript.

## Supplementary Material

Additional file 1 **The positive control for SMILE and Zhangfei protein was analysed by western blotting using a CREBZF-specific antibody.** Results are representative of at least three independent experiments with similar results. MCF7, human breast adenocarcinoma cell line. 293 T, human embryonic kidney cell line. Click here for file
