# Supplementary figures and images for: CREBZF expression and hormonal regulation in the mouse uterus
Source: Reprod Biol Endocrinol. 2013 Dec 10;11:110. doi: 10.1186/1477-7827-11-110 (PMC3878900; doi:10.1186/1477-7827-11-110)

293T      MCF7

62 kDa

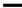

45 kDa

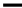

30 kDa

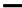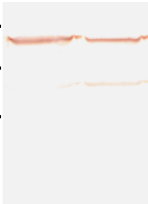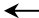

SMILE

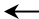

Zhangfei

Supplement: Additional file 1 — The positive control for SMILE and Zhangfei protein was analysed by western blotting using a CREBZF-specific antibody. Results are representative of at least three independent experiments with similar results. MCF7, human breast adenocarcinoma cell line. 293 T, human embryonic kidney cell line. [file 1477-7827-11-110-S1.pdf]
